# Machine learning for pan-cancer classification based on RNA sequencing data

**DOI:** 10.3389/fmolb.2023.1285795

**Published:** 2023-11-10

**Authors:** Paula Štancl, Rosa Karlić

**Affiliations:** Bioinformatics Group, Division of Molecular Biology, Department of Biology, Faculty of Science, University of Zagreb, Zagreb, Croatia

**Keywords:** cancer of unknown primary, tissue of origin, cancer classification, machine learning, RNA sequencing

## Abstract

Despite recent improvements in cancer diagnostics, 2%-5% of all malignancies are still cancers of unknown primary (CUP), for which the tissue-of-origin (TOO) cannot be determined at the time of presentation. Since the primary site of cancer leads to the choice of optimal treatment, CUP patients pose a significant clinical challenge with limited treatment options. Data produced by large-scale cancer genomics initiatives, which aim to determine the genomic, epigenomic, and transcriptomic characteristics of a large number of individual patients of multiple cancer types, have led to the introduction of various methods that use machine learning to predict the TOO of cancer patients. In this review, we assess the reproducibility, interpretability, and robustness of results obtained by 20 recent studies that utilize different machine learning methods for TOO prediction based on RNA sequencing data, including their reported performance on independent data sets and identification of important features. Our review investigates the strengths and weaknesses of different methods, checks the correspondence of their results, and identifies potential issues with datasets used for model training and testing, assessing their potential usefulness in a clinical setting and suggesting future improvements.

## 1 Introduction

Cancer is the leading cause of death worldwide, and the overall burden of cancer incidence and mortality is expected to increase due to the growing population, aging inhabitants, and changes in the prevalence of risk factors (Sung et al., 2021). Key factors in reducing cancer incidence and improving the survival of cancer patients include prevention, early detection, and the availability of appropriate treatment. Despite the recent advances in cancer diagnostics, cancers of unknown primary (CUP), in which the tissue-of-origin (TOO) cannot be identified at the time of presentation, still constitute approximately 2%–5% of all malignancies. Since the primary site of cancer determines the choice of optimal treatment, CUP patients present a significant clinical challenge with limited treatment options. Although a fraction of CUP patients can be assigned the correct TOO and receive appropriate treatment based on the analysis of clinical, imaging, or histopathological data, this is still not the case for the majority of CUP patients, who then face a less favorable prognosis (Binder et al., 2018; Rassy and Pavlidis, 2020).

The development of array- and next-generation sequencing (NGS)-based whole-genome profiling techniques has enabled the rapid molecular characterization of cells or tissues, inspiring the establishment of several large-scale cancer genomics initiatives, such as ICGC and TCGA (International Cancer Genome Consortium et al., 2010; Weinstein et al., 2013). These initiatives aim to describe the genomic, epigenomic, and transcriptomic characteristics of a large number of individual patients with multiple types of cancer. The unprecedented volume of data produced by these techniques has led to the introduction of various methods that use machine learning to predict the TOO for cancer patients. This is most frequently done by analyzing somatic alterations, gene expression, microRNA expression, or DNA methylation of cancer samples. Most of the TOO prediction tools currently used in clinical practice are based on qRT-PCR or microarray measurements of different features of preselected genes. The accuracy of such tools typically falls within the range of 54%–100%. While several clinical trials have demonstrated improved overall survival of CUP patients who received tumor-type-specific therapy based on predicted TOO, inconsistencies in the results of randomized and non-randomized trials suggest that there are opportunities for improvement in this area of research (Conway et al., 2019; Rassy et al., 2020).

Various methods that utilize machine learning to predict the TOO based on NGS data have been developed recently, although the clinical use of such methods is still limited (Swanson et al., 2023). Advantages of molecular characterization using NGS approaches, in comparison to array-based techniques, include increased specificity and sensitivity, a broader dynamic range, and whole-genome coverage. Indeed, several recent reviews on the topic of machine learning and deep learning for cancer classification have reported the excellent performance of methods that utilize NGS data (Tufail et al., 2021; Alharbi and Vakanski, 2023). However, the high dimensionality, sparsity, and heterogeneity of input data, as well as dataset imbalance, could lead to issues with overfitting and high variance of the trained models.

In this minireview, we aim to assess the reproducibility, interpretability, and robustness of different NGS-based TOO prediction methods, including reported performance on independent data sets and identification of important features. Since the accuracy of the models depends on the input data type (Conway et al., 2019; Rassy et al., 2020), we have limited our survey to tools developed exclusively on RNA sequencing (RNA-Seq) data. This decision was made considering that they represent the majority of studies. We focused on studies developed on pan-cancer data that include at least nine different cancer types in training data. Our review investigates the strengths and weaknesses of different methods, checks the correspondence of their results, and provides an assessment of their potential usefulness in a clinical setting.

## 2 Machine learning in cancer of unknown primary classification based on RNA sequencing

We have conducted a literature search to identify studies that used RNA-Seq data to train machine learning models for cancer classification and TOO prediction. Studies that relied exclusively on miRNA sequencing were excluded from further analysis. Based on the aforementioned criteria, we selected 20 recently published studies for analysis.

The majority of studies were based on deep learning, using neural networks of different architectures (Lyu and Haque, 2018; Azarkhalili et al., 2019; De Guia et al., 2019; He et al., 2020b; Mostavi et al., 2020; Zhao et al., 2020; Vibert et al., 2021; Divate et al., 2022; Hong et al., 2022; Jones et al., 2022; Moiso et al., 2022). Several studies utilized ensemble learning methods, in which the final prediction is a combination of multiple predictors (Grewal et al., 2019; He et al., 2020a; Ramroach et al., 2020; Chen et al., 2021; Liu et al., 2021). Bavafaye Haghighi et al. (2019) and Galea et al. (2017) classified different cancer types using hierarchical classification, a ‘top-down’ classification approach in which classification models are trained at each level of the hierarchy. Additionally, some authors employed simpler machine learning methods, such as k-nearest neighbors (Bagge et al., 2018) or stepwise logistic regression (Wei et al., 2014). Several studies tested their results against additional classification methods (Azarkhalili et al., 2019; De Guia et al., 2019; Grewal et al., 2019; Ramroach et al., 2020; Hong et al., 2022), or compared the performances of various deep learning architectures (Mostavi et al., 2020; Zhao et al., 2020; Vibert et al., 2021). In cases where multiple approaches were used in a single study, we limited our analysis to the best-performing model.

The models were trained on datasets comprising 9 to 40 cancer types (with a median number of 32 cancer types), and the number of sample points used for training ranged from 1,960 to 20,918 (with a median number of 10,116 samples). All of the selected studies trained their models on either TCGA or ICGC data, with some studies including cancer sequencing data produced in-house (Wei et al., 2014) or data from healthy tissues (Azarkhalili et al., 2019; Grewal et al., 2019; Mostavi et al., 2020; Vibert et al., 2021).

## 3 Performance of models for tissue-of-origin prediction

We have compared the classification accuracy of various models, which we defined as the number of correct predictions divided by the number of total predictions. In the cases where the classification accuracy was not reported, we have calculated it from the results described in the original publication. Cases where no prediction could be made were counted as incorrect predictions. Accuracy was the most commonly used measure of predictive performance in the studies surveyed in this minireview. While this measure can be influenced by class-imbalanced data, the influence of training dataset composition on measures of predictive performance is outside of the scope of this minireview.

**FIGURE 1:**
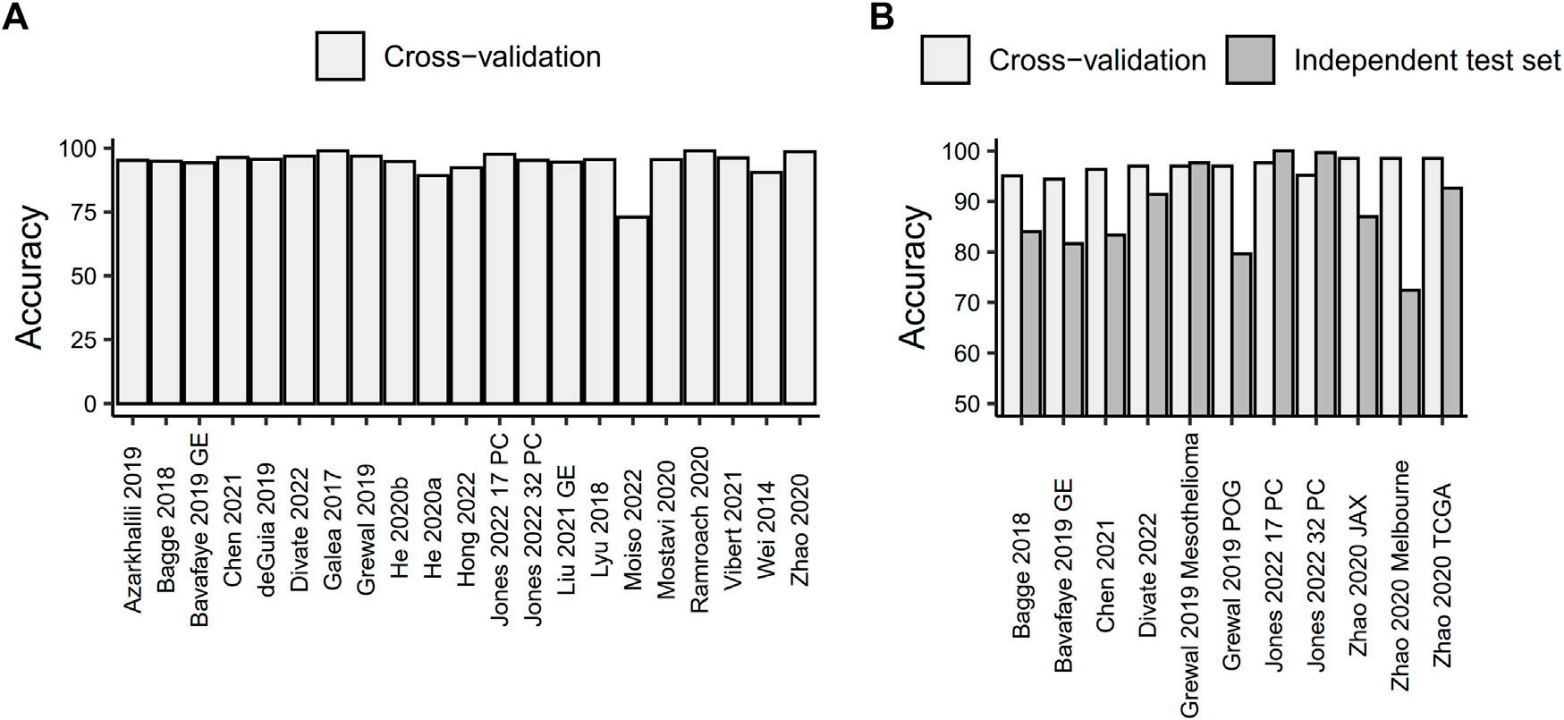
Prediction accuracy of machine learning models for tissue-of-origin prediction based on RNA sequencing data. **(A)** Cross-validated prediction accuracy for all models. **(B)** Comparison of cross-validated prediction accuracy and accuracy measured on an independent test set.

In general, the analyzed models achieve high cross-validation prediction accuracy in the range of 73%–99% (with a median cross-validation accuracy of 95.5%; Figure 1A). This accuracy does not seem to depend on the number of training points (Spearman’s correlation coefficient *ρ* = 0.0591, *p*-value = 0.7989). The prediction accuracy varies by tumor type, with some tumor types being more frequently mispredicted. Patterns of more frequent misclassifications among groups of cancers arising from the same organ (e.g., kidney renal clear cell carcinoma, kidney renal papillary cell carcinoma and kidney chromophobe carcinoma, or lung adenocarcinoma and lung squamous cell carcinoma), and/or among cancers represented by a small number of samples in the training set (e.g., cholangiocarcinoma, which is frequently predicted as liver hepatocellular carcinoma and *vice versa*), as noted in multiple studies (Bagge et al., 2018; Lyu and Haque, 2018; Bavafaye Haghighi et al., 2019; De Guia et al., 2019; Grewal et al., 2019; Zhao et al., 2020; Vibert et al., 2021; Divate et al., 2022; Jones et al., 2022; Moiso et al., 2022). All of this implies that the distribution of different cancer types in the training set is one of the key factors contributing to the prediction accuracy of the model.

Out of the studies included in this minireview, only seven tested the performance of the developed models on an independent test set. Overall, the predictive accuracy of models on independent test data was lower than the cross-validation accuracy calculated on the data used for training the model, with only two studies attaining a prediction accuracy on an independent test set that was comparable to or higher than the one obtained with cross-validation (Figure 1B). Jones et al. (2022) used 277 primary kidney cancer samples from the CPTAC consortium to test their convolutional neural network-based models, which were trained on 17 or 32 primary cancer types. They achieved prediction accuracies of 100% and 99.63%, respectively. An ensemble of neural networks developed by Grewal et al. (2019) correctly predicted the primary cancer type for 96.73% of 211 samples from the independent Genentech Mesothelioma dataset. However, both of those models were tested on independent data comprising a single primary cancer type, which may not necessarily reflect the putative prediction accuracy on a pan-cancer dataset. In fact, the accuracy of the Grewal et al. (2019) model decreased to 79.60% when tested on an additional independent test set of 201 patients spanning 26 different cancer types. These patients were sequenced as part of the Personalized OncoGenomics project at the BC Cancer Agency and presented with metastatic disease that no longer responds to treatment. The remaining studies used independent test sets consisting of 5–18 cancer types and showed a reduction in prediction accuracy ranging from 5.8% to 26.47% compared to cross-validation (with a mean reduction in accuracy of 13.32%). Out of these, two studies used metastatic samples (Bavafaye Haghighi et al., 2019; Zhao et al., 2020), two used a mixture of primary and metastatic samples (Bagge et al., 2018; Divate et al., 2022) and one did not report the exact source of the independent test set (Chen et al., 2021).

Interestingly, when testing the accuracy of the same model on test sets composed of both primary and metastatic samples, metastatic samples showed a lower prediction accuracy. For example, Divate et al. (2022) reported 88.10% accuracy for metastatic samples compared to 92.13% for primary samples. Bagge et al. (2018) found 53.84% accuracy for metastatic samples compared to 96.67% for patient-derived xenografts of primary cancer and 100% for primary cancer. However, this difference could also be attributed to the distribution of the primary cancer types from which the metastases arose in the test set and their potential underrepresentation in the training data.

Furthermore, Zhao et al. (2020) used three different independent test sets in their research, including two datasets obtained by RNA-Seq of formalin-fixed paraffin-embedded (FFPE) metastatic cancer samples. The FFPE-based datasets showed a lower prediction accuracy (86.96% for the JAX clinical dataset, which included 23 samples across 6 cancer types, and 72.46% for the Melbourne clinical dataset, which encompassed 69 samples across 18 cancer types) compared to RNA-Seq conducted on fresh frozen tissue samples (92.64% for the TCGA, which included 394 samples across 11 cancer types). This suggests that the methods used for tissue processing and storage could impact the results obtained from different sample types.

## 4 Identification of informative gene sets for cancer classification

Several methods employed feature selection approaches to identify the most informative sets of genes for TOO prediction using different strategies to choose relevant genes. Two studies utilized various subsets of top-selected genes based on the Gini index from Random Forest models (He et al., 2020a; Ramroach et al., 2020). Similarly, neural network models selected a certain number of top-ranked genes, either based on the highest weights for the tumor class (Grewal et al., 2019) or by calculating Shapley additive explanation values in deep neural network models (Divate et al., 2022). Wei et al. (2014) employed univariate transcript analysis with stepwise logistic regression to select the top N transcripts. Their approach achieved high AUC prediction values using only a smaller number of genes. However, the results of two randomizations of the feature selection process show that, although the number of selected genes was similar, the correspondence between the two randomizations was low, with a cosine similarity of 0.53. Another approach was to select highly variable genes across various cancers and either use the top N genes for modeling (Moiso et al., 2022) or perform dimensionality reduction on them before training machine learning models like variational autoencoders (Vibert et al., 2021). Furthermore, feature selection could be performed by selecting the most important genes in each cancer type. Zhao et al. (2020) did this by selecting the top N differentially expressed genes in each cancer type to create a unique list of genes. A similar approach, based on the Pearson correlation algorithm, was employed by He et al. (2020b). Other approaches involved the selection of genes based on prior knowledge, such as choosing clinical oncopanel genes tested in routine clinical cancer care (Moiso et al., 2022) or a catalog of somatic mutations in cancers (COSMIC) list of genes harboring somatic mutations (Grewal et al., 2019).

**FIGURE 2:**
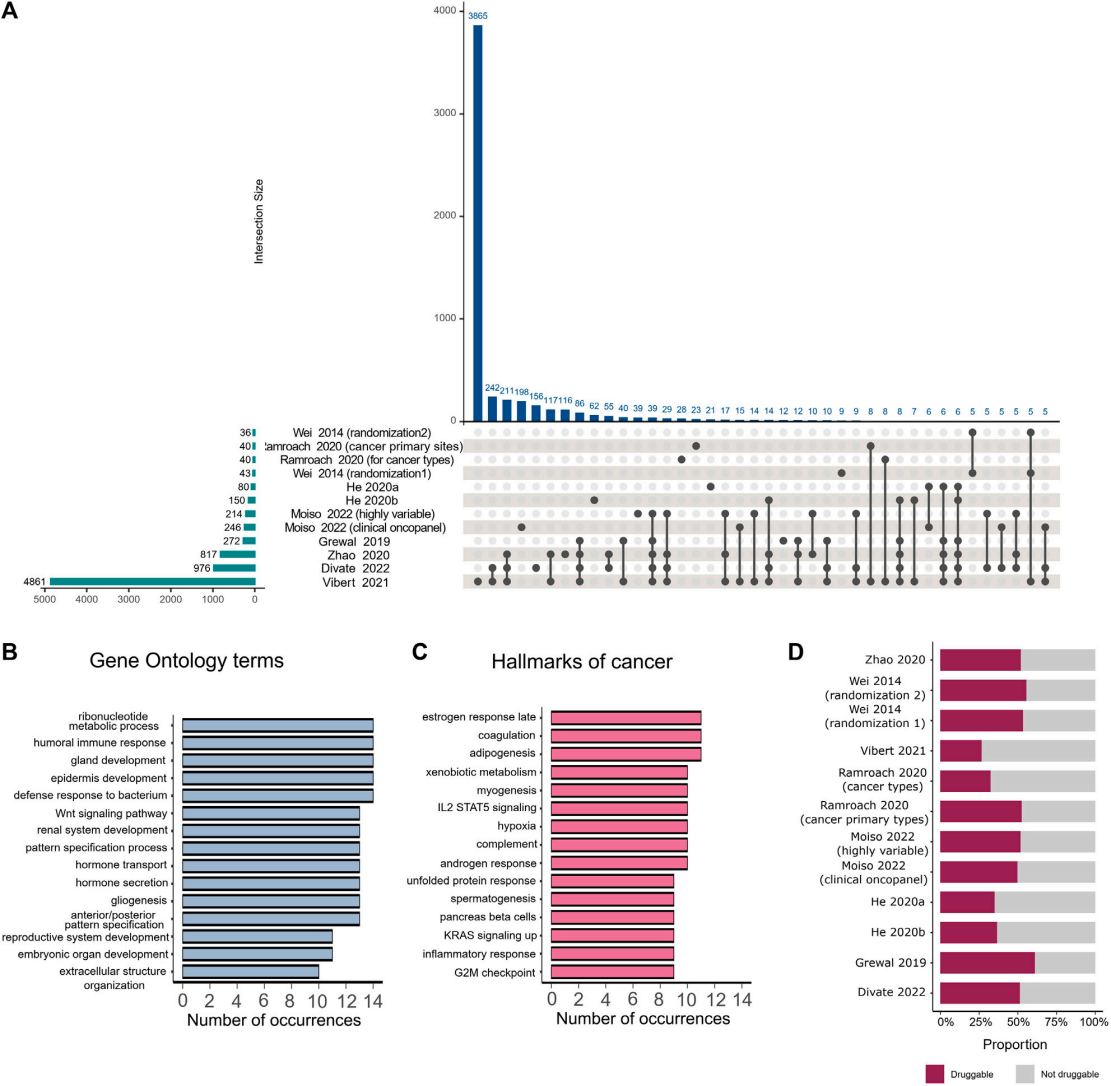
Comparison of important genes identified by various feature selection methods. **(A)** The intersection plot shows overlaps between genes detected as important by different studies. Numbers of genes identified by individual studies are displayed as horizontal bars on the lower left corner of the image. Intersection sizes are shown as individual bars on the top of the plot. Specific studies involved in each intersection are identified with connected solid black circles under the vertical bars, with unconnected circles representing genes that are detected exclusively in the corresponding study. **(B)** Number of occurrences of different Gene Ontology terms in studies that employed feature selection. **(C)** Number of occurrences of different hallmarks of cancers in studies that employed feature selection. **(D)** Proportion of druggable genes among all genes that were selected by feature selection in each study.

To estimate the correspondence of informative gene sets identified by various studies, we extracted gene lists provided by the studies that employed feature selection on the primary dataset. Studies selected between 36 and 4,861 unique genes, with the highest number of overlapping genes found between gene lists identified by Divate et al. (2022), Zhao et al. (2020), and Vibert et al. (2021), which collectively chose the highest number of genes among all the analyzed studies (Figure 2A). No genes were found to be selected by all studies.

We mapped the extracted gene lists to hallmarks of cancer (Dolgalev, 2022) and Gene Ontology terms (Wu et al., 2021) and analyzed the number of occurrences of each hallmark or term among the different studies. The majority of selected genes belonged to specific pathways such as embryonic organ development, gland development, hormone metabolic processes, reproductive system development, and morphogenesis of an epithelium. These pathways were observed in more than 10 studies (Figure 2B). The most frequently occurring hallmarks of cancer, found in the majority of studies, were reproductive system hallmarks such as estrogen late response, androgen response, and spermatogenesis (Figure 2C). We also examined which genes in each study were druggable according to The Drug Gene Interaction Database (Beerenwinkel et al., 2016; Wagner et al., 2016), following the method proposed by Ramroach et al. (2020), and found that all studies selected at least 25% druggable genes (Figure 2D).

We further examined genes proposed by different studies as potential cancer signatures. Well-known prostate cancer signatures, including *KLK3*, a serine protease used as a serum marker in prostate cancer screening and disease monitoring, and *PRAC*, a highly expressed gene in prostate cancer (Edwards et al., 2005), have been selected by 7 and 3 studies, respectively. Another protein selected by 7 studies is the lung biomarker *NAPSA*, an aspartic proteinase expressed in type II pneumocytes. Its expression can be used to distinguish pulmonary lesions originating from primary lung adenocarcinoma or other primaries (Ueno et al., 2003). Additionally, 5 other studies identified *IGFBP1*, a hepatocyte-derived secreted protein required for normal liver regeneration by inhibiting proapoptotic signals (Borlak et al., 2005), as an important feature, particularly for the identification of hepatocellular carcinoma, in which it is overexpressed.

Wei et al. (2014) identified additional potential cancer signatures not previously associated with the cancer types of interest. Some of those genes, such as *DPYS*, were also identified by three more recent studies (Zhao et al., 2020; Vibert et al., 2021; Divate et al., 2022) as important for TOO prediction, especially for kidney cancer. Other genes proposed by Wei et al. (2014), such as the potential new ovarian biomarker *BEST1* and new prostate and gastric biomarker *SI*, were either not found by other studies or were only identified by Vibert et al. (2021), despite having implications in other cancer types.

Ramroach et a. (2020) stated that the majority of the top 40 genes selected by their study belong to the olfactory receptor family, keratin-associated proteins, or the defensin beta family. Interestingly, although it was claimed that the olfactory receptor family plays a significant role in cancer, those genes were not detected by any other study. Genes belonging to keratin-associated proteins were only identified by Vibert et al. (2021). From the defensin beta family, only the *DEFB1* gene was detected by four different studies (Grewal et al., 2019; Zhao et al., 2020; Vibert et al., 2021; Divate et al., 2022), however, this particular gene was not included in the top 40 genes selected by Ramroach et al. (2020).

## 5 Conclusion and future improvements

In this minireview, we analyzed 20 recent studies that employed machine learning to predict the TOO of cancers based on NGS data. Our goal was to assess their performance, reproducibility, interpretability, and robustness. We found that all of the analyzed methods exhibited very high prediction accuracy, ranging from 73% to 99%. This performance represents an improvement over currently used microarray-based methods, which have a prediction accuracy of 54%–100% (Conway et al., 2019; Rassy et al., 2020). These findings suggest that these machine learning approaches have the potential to bring about significant advancements in the diagnosis and treatment of cancers of unknown primary.

However, while the overall prediction accuracy of the models is high, it varies by tumor type, with tumors originating from the same organ or tumors that are underrepresented in the training set being more frequently mispredicted. This suggests that the accuracy depends more on the composition of the training set than on the method used for training the model. Researchers should, therefore, aim to assemble balanced datasets for model training and include as many samples of rare and underrepresented cancers as possible.

Furthermore, most of the analyzed studies did not employ an independent test set, and the ones that did mostly showed a reduction in accuracy, especially for test sets obtained from metastatic patients or FFPE samples. Since CUP patients are, by definition, metastatic patients and FFPE tissues are still the most commonly available sample type for RNA-Seq, due to the cost-effectiveness of storage (Zhao et al., 2019), both metastatic samples from as many cancer types and FFPE samples should be included in independent test sets to support the claims of potential clinical use of NGS-based approaches to cancer classification. Additional factors, such as data quality and tumor purity, should also be investigated to determine their potential impact on model accuracy.

Identification of features important for prediction, implemented by several of the analyzed studies, could lead to novel biomarker discovery and discovery of genes whose expression is dysregulated in cancer, expand our current knowledge of mechanisms of cancer development and progression, identify potential actionable targets, and inspire novel treatment strategies. Indeed, most of the studies that employed feature selection identified at least 25% of actionable targets among their set of selected genes and showed that some of those genes are already known cancer signature genes. However, the overlap of gene lists provided by different studies is quite low, indicating that these results should be interpreted with caution. It is important to note that the majority of the analyzed studies employed filter methods, which select relevant features based on their intrinsic characteristics. Some used wrapper methods, where features are added or removed iteratively and scored based on their impact on the machine learning model’s performance, or embedded approaches, which combine properties of both filter and wrapper methods.

Filter methods, while computationally less demanding, typically do not consider the subsequent classification model, often resulting in inferior performance compared to wrappers. In contrast, wrappers can be susceptible to overfitting and sensitive to parameter adjustments (Zanella et al., 2022). Recently, various nature-inspired algorithms, such as those based on swarm intelligence and evolutionary principles, have been applied as metaheuristic search methods for wrapper-based feature selection problems and show significant potential in the identification of relevant genes for cancer classification using microarray gene expression measurements (Pham and Raahemi, 2023; Yaqoob et al., 2023).

For example, in a study using ten microarray datasets for cancer classification, Gene Selection Programming (Alanni et al., 2019), a method for selecting relevant genes based on Gene Expression Programming (Ferreira, 2002), demonstrated the highest accuracy and the fewest selected genes in the majority of cases, outperforming swarm-based algorithms and more traditional methods like support vector machines. This suggests that the application of such methods to RNA-Seq datasets could lead to more accurate and robust gene selection for cancer classification. Furthermore, the availability of additional sequencing data and the investigation of possible biases that could influence modeling results could further enhance the clinical applicability of methods described in this minireview and similar tools.
